# Overexpression of protein phosphatase 5 in the mouse heart: Reduced contractility but increased stress tolerance – Two sides of the same coin?

**DOI:** 10.1371/journal.pone.0221289

**Published:** 2019-08-19

**Authors:** Ulrich Gergs, Tina Jahn, Franziska Werner, Carolin Köhler, Friedrich Köpp, Claudia Großmann, Joachim Neumann

**Affiliations:** 1 Institut für Pharmakologie und Toxikologie, Medizinische Fakultät, Martin-Luther-Universität Halle-Wittenberg, Halle (Saale), Germany; 2 Julius-Bernstein-Institut für Physiologie, Medizinische Fakultät, Martin-Luther-Universität Halle-Wittenberg, Halle (Saale), Germany; University of Cincinnati College of Medicine, UNITED STATES

## Abstract

The pathophysiological mechanisms of sepsis-induced cardiac dysfunction are largely unknown. The Toll-like receptor 4 (TLR4) is expressed in cardiac myocytes and is involved in bacterial endotoxin-mediated inflammatory disorders. TLR4 signaling leads to activation of the nuclear factor kappa B followed by increased expression of cytokines. Several protein phosphatases including PP2Cβ, PP2A or PP1 are known to act as regulators of this signaling pathway. Here, we examined the role of PP5 for the inflammatory response to the bacterial endotoxin lipopolysaccharide in the heart using a transgenic mouse model with cardiac myocyte directed overexpression of PP5. In these transgenic mice, basal cardiac contractility was reduced, *in vivo* as well as *in vitro*, but LPS-induced cardiac dysfunction was less pronounced compared to wild type mice. Quantitative RT-PCR suggested an attenuated NF-κB signaling in the heart and cardiac expression of heat shock protein 25 (HSP25) was increased in PP5 transgenic mice. From our data we assume that PP5 increases stress tolerance of cardiac myocytes by downregulation of NF-κB signaling and upregulation of HSP25 expression.

## Introduction

The bacterial endotoxin lipopolysaccharide (LPS) acts via Toll-like receptor 4 (TLR4) and is responsible for inflammatory disorders including acute heart failure [1]. TLR4 is expressed in cardiac myocytes and is involved in LPS-mediated cardiac dysfunction [2]. The TLR4 signaling pathway starts with a protein complex for LPS recognition outside of the cell. This complex includes the LPS-binding protein (LBP), the cluster of differentiation 14 (CD14) protein and the adapter protein MD2. Its formation is necessary for the dimerization and activation of TLR4, initiating the intracellular signaling cascade [1]. One important branch of this signaling pathway ends in activation of the nuclear factor kappa B (NF-κB). In the heart, increased transcriptional activity of NF-κB initiates an inflammatory response (e.g. expression of cytokines like IL-1, IL-6, TNFα) finally leading to decreased contractility [3].

An important step in NF-κB activation includes the phosphorylation of the NF-κB inhibitor IκB by the IκB kinase complex (IKKα/β/γ). Phosphorylated IκB dissociates from NF-κB leading to its activation [4]. Several protein phosphatases (PP) are known to act as negative regulators of the IKK complex. These are PP2Cβ, PP2A or PP1 and more recently also PP5 was found to negatively modulate NF-κB activity [5]. It was reported that G4-1, also known as G5PR, a B” regulatory subunit of PP2A can interact with IKKβ and by this way can recruit also PP5 to the IKK complex leading to inactivation of IKKβ [5].

Protein phosphatase 5 (PP5) is a member of the PPP family of serine/threonine phosphatase like PP1, PP2A or PP2B but with the unique feature that catalytic, regulatory and targeting functions are combined on one protein [6]. Also in contrast to other protein phosphatases are the three N-terminal tetratricopeptide repeat (TPR) domains, responsible for protein-protein interactions and for an inhibitory interaction with the C-terminus leading to low basal phosphatase activity [7]. Via its TPR domains, PP5 binds other proteins of which heat shock protein 90 (HSP90) is the best known. HSP90 mediates the interaction of PP5 for example with estrogen and glucocorticoid receptors [7,8]. Additionally, PP5 plays a role in cellular proliferation where p53 phosphorylation and the anaphase-promoting complex are involved in PP5-dependent regulations [9]. Moreover, PP5 acts as negative regulator of the heat shock factor (HSF1), a transcription factor needed for up-regulation of heat shock proteins like HSP70 and HSP90 [10].

Little is known about cardiac functions of PP5. Recently, it was reported that S100 proteins can modulate PP5 functions [11] and S100A1 that amongst others interacts with the cardiac ryanodine receptor and the Ca^2+^ ATPase of the sarcoplasmic reticulum (SERCA), plays an important role in the inflammatory response in cardiomyocytes [12,13]. We have previously generated and described transgenic mice with cardiac specific overexpression of PP5 and demonstrated that PP5 serves as a modulator of cardiac function [14]. Moreover, PP5 in association with HSP90 interacts with the sarcomeric mechanosensor complex and regulates titin phosphorylation and function at the myofilaments of cardiac myocytes [15]. Here, we hypothesized that PP5 also plays a role in the inflammatory response to LPS in the heart. Therefore, we used our transgenic mouse model and monitored cardiac function and gene expression after LPS injection.

Parts of this work have been published in abstract form [16–19].

## Materials and methods

### Transgenic mice

Transgenic mice with cardiomyocyte-specific overexpression of PP5 were generated utilizing a mouse cardiac α-myosin heavy chain promoter expression cassette containing the cDNA of rat PP5 along with 483 base pairs of the 3’ untranslated region as described previously [14]. Hearts from transgenic mice showed a 4.5 fold overexpression of PP5 on protein level. Morphology of the heart was unchanged but on functional level a reduced ventricular contractility under basal conditions and a reduced response to β-adrenergic stimulation was found compared to wild type mice. Moreover, in isolated transgenic cardiac myocytes, the L-type Ca^2+^ current was reduced. For the experiments, mice from 6 to 8 months old of each gender were used. Where indicated, intraperitoneal injection of 25 mg/kg LPS from E. coli (O55:B5; Sigma-Aldrich, Munich, Germany) was performed. As a control, isotonic NaCl solution (NaCl) was injected. The investigation conforms with the Guide for the Care and Use of Laboratory Animals published by the National Research Council (US) 2011 [20]. The protocol was approved by the local committee on the ethics of animal experiments of the state Sachsen-Anhalt (Permit Number: 42502-02-691 MLU). Echocardiography was performed under isoflurane anesthesia and pentobarbital was used for euthanasia of mice. All efforts were made to minimize suffering.

### Real time PCR

Total RNA was isolated using the TRIzol reagent (Invitrogen, Fisher Scientific, Schwerte, Germany) according to the manufacturer’s instructions. Subsequently, reverse transcription was performed using the Maxima First Strand cDNA Synthesis Kit (Fisher Scientific, Schwerte, Germany) according to the manufacturer’s instructions. During cDNA synthesis, remaining DNA was digested with DNase I. Reverse transcription was performed with 3–5 μg RNA and a mixture of oligo(dT)18 and random hexamer primers. As a control, each RNA sample was also analyzed without reverse transcription (NRT). Finally, cDNA samples were diluted to a volume corresponding to 0.1 μg RNA per μl. Real time PCR amplification and detection was performed with the BioRad CFX Connect system using the iTaq SYBR Green kit (Bio-Rad Laboratories, Munich, Germany) according to the manufacturer’s instructions. The relative expression of the genes of interest was calculated according to the 2^−ΔΔCT^ method [21], using the 18S signal or the GAPDH signal for normalization. Primers were either developed with the software Discovery Studio Gene v1.5 (Accelrys, Cambridge, UK) or were derived from the literature [22–27]. Primer sequences are summarized in S1 Table.

### Western blot analysis

For Western blot analysis, ventricular homogenates were prepared and aliquots of 50 to 200 μg protein were loaded per lane as described previously [28]. Protein loading was monitored by Ponceau staining of the nitrocellulose membranes. Bands were detected using enhanced chemifluorescence (ECF) and a Typhoon 9410 Variable Mode Imager (GE Healthcare, Freiburg, Germany). Signals were quantified with the ImageQuant TL software (GE Healthcare, Freiburg, Germany). The list of primary antibodies used is summarized in S2 Table. Corresponding secondary antibodies conjugated to alkaline phosphatase were purchased from Sigma-Aldrich (Munich, Germany).

### Echocardiography

Transthoracic echocardiographic measurements in spontaneously breathing mice were performed under anesthesia with 1.5% isoflurane using a Vevo 2100 system equipped with a MS 550D transducer (Visual Sonics, Toronto, Canada). Two-dimensional images and M-mode tracings were recorded from the parasternal long axis view. Cardiac dimensions were measured and the ejection fraction of the hearts was calculated [29].

### Work-performing heart preparations

Work-performing heart preparations were utilized as described previously [14]. Heart rate, aortic pressure, left intraventricular pressure (systolic, diastolic, and end diastolic), and atrial pressure were measured and monitored continuously. The first derivative of left intraventricular pressure (+dP/dt and–dP/dt) was calculated with a computer program (PowerLab; ADInstruments, Spechbach, Germany).

### Statistics

Data shown are means ± S.E.M. Statistical significance was estimated by analysis of variance (ANOVA) followed by Bonferroni’s posttest or using the student’s t-test as appropriate. A p-value < 0.05 was considered significant. Statistical evaluation was conducted with GraphPad Prism 5.0 (GraphPad Software, San Diego, California, USA), which was also used to produce graphs.

## Results

Adult transgenic mice (PP5) and wild type littermates (WT) of both sexes were used at the age of 6 to 8 month. They were divided in two experimental groups. First, for measurement of acute effects, a single dose of LPS (25 mg/kg body weight) or isotonic NaCl solution as control were applied for 7 h. Cardiac function of each mouse was monitored *in vivo* before and 3 h and 7 h after LPS (or NaCl) application by echocardiography. After 7 h, hearts were removed for biochemical analysis. Second, for measurement of effects over a prolonged time period, a single dose of LPS (NaCl) was applied and after 3 days, cardiac function was measured in isolated perfused hearts followed by biochemical analysis.

**Fig 1:**
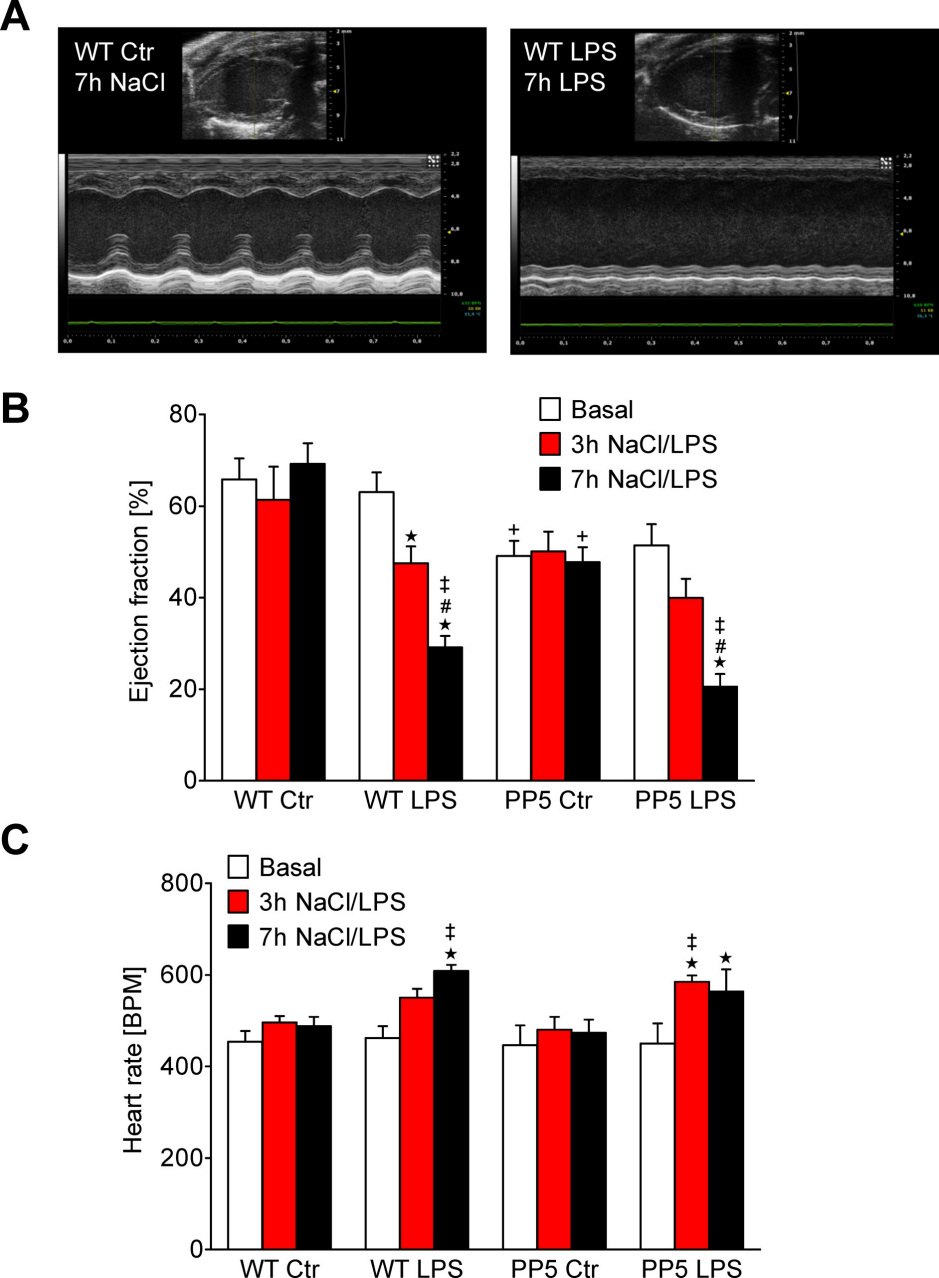
Echocardiography. Measurement of echocardiographic parameters of wild type (WT) and PP5 overexpressing mice before (basal) and 3 h and 7 h after intraperitoneal application of LPS (25 mg/kg) or NaCl solution as control. (**A**) Representative images (B- and M-mode) illustrating the effect of LPS, (**B**) ejection fraction (**C**) heart rate ★p < 0.05 vs. basal; #p < 0.05 vs. 3 h LPS; ‡p < 0.05 vs. Ctr; +p < 0.05 vs. WT.

In the first experimental group, body weight and heart weight were not different between WT and PP5 mice and, moreover, unchanged after 7 h LPS compared to control (S3 Table). Cardiac contractility, monitored by echocardiography (Fig 1), was already reduced in PP5 under basal conditions as demonstrated by a reduced ejection fraction (EF) in Fig 1B. LPS application, time-dependently reduced the EF in WT and PP5 mice (Fig 1B). This effect was more pronounced in WT and finally, after 7 h, EF was no longer different between WT and PP5. Heart rate was not different between WT and PP5 mice and increased over the course of 7 h LPS treatment in both, WT and PP5 mice (Fig 1C). In the second experimental group, three days after LPS application, body weight of both, WT and PP5, was reduced from 33.6 ± 2.6 g to 30.1 ± 2.6 g for WT and from 34.9 ± 1.5 g to 30.4 ± 1.4 g for PP5 mice (p < 0.05). In the control (NaCl) groups, body weights were completely unchanged. Heart weights remained unchanged for all conditions. As a sign of successful induction of sepsis by LPS, the spleen weight was increased by 89% in both, WT and PP5 (p < 0.05) compared to control mice (S4 Table).

**Fig 2:**
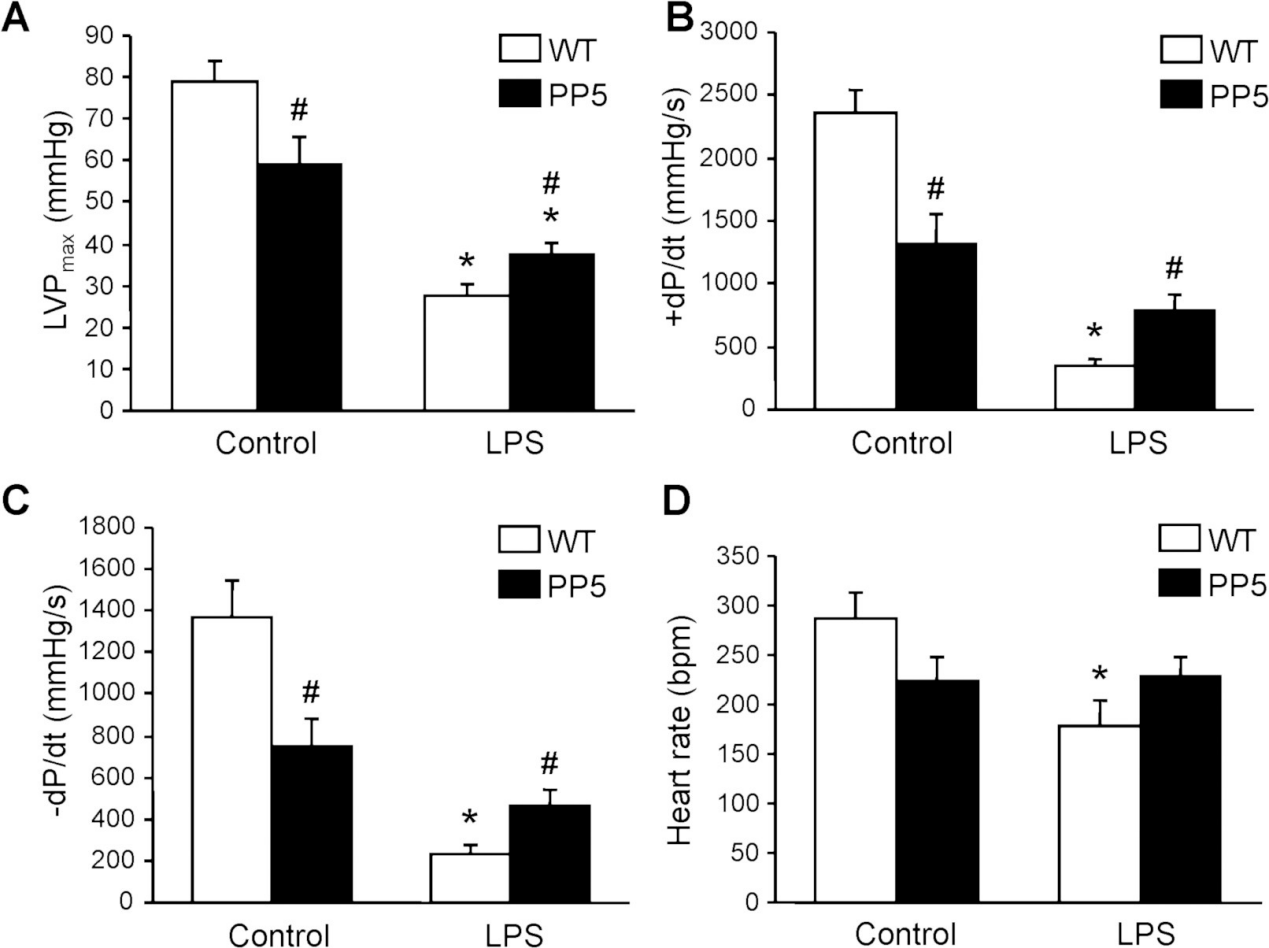
Isolated perfused hearts. Working heart preparations of wild type and PP5 overexpressing mouse hearts 3 days after intraperitoneal application of LPS (25 mg/kg) or NaCl solution as control. (**A**) Maximum left ventricular pressure (LVPmax), (**B**) maximum rate of left ventricular pressure development (+dP/dt), (**C**) maximum rate of left ventricular pressure decline (-dP/dt), (**D**) heart rate. ★p < 0.05 vs. control; #p < 0.05 vs. WT.

Cardiac contractility was estimated independently from humoral and neuronal influence using work-performing heart preparations and the results are presented as maximum left ventricular pressure (Fig 2A) as well as maximum rate of left ventricular pressure development or decline (Fig 2B and 2C). Again, basal contractility of PP5 hearts was reduced compared to WT (control group in Fig 2A–2C). LPS deteriorated cardiac function in both, PP5 and WT, but in relative terms to a much lesser extent in PP5 compared to WT (Fig 2A–2C). Interestingly, heart rate of isolated perfused hearts was lowered by LPS treatment in WT but not in PP5 (Fig 2D). The opposite was found in living animals by echocardiography where LPS treatment increased heart rate in WT and PP5 mice (Fig 1B). The *in vivo* and the *in vitro* data indicate that PP5 mainly influences cardiac contractility but less the heart rate. Especially the differences in heart rate between echocardiography and isolated heart measurements demonstrate the need of *in vivo* experiments to estimate the importance of an effect for the whole organism. On the other hand, in vitro studies are very often the only way to assess the underlying mechanism.

**Fig 3:**
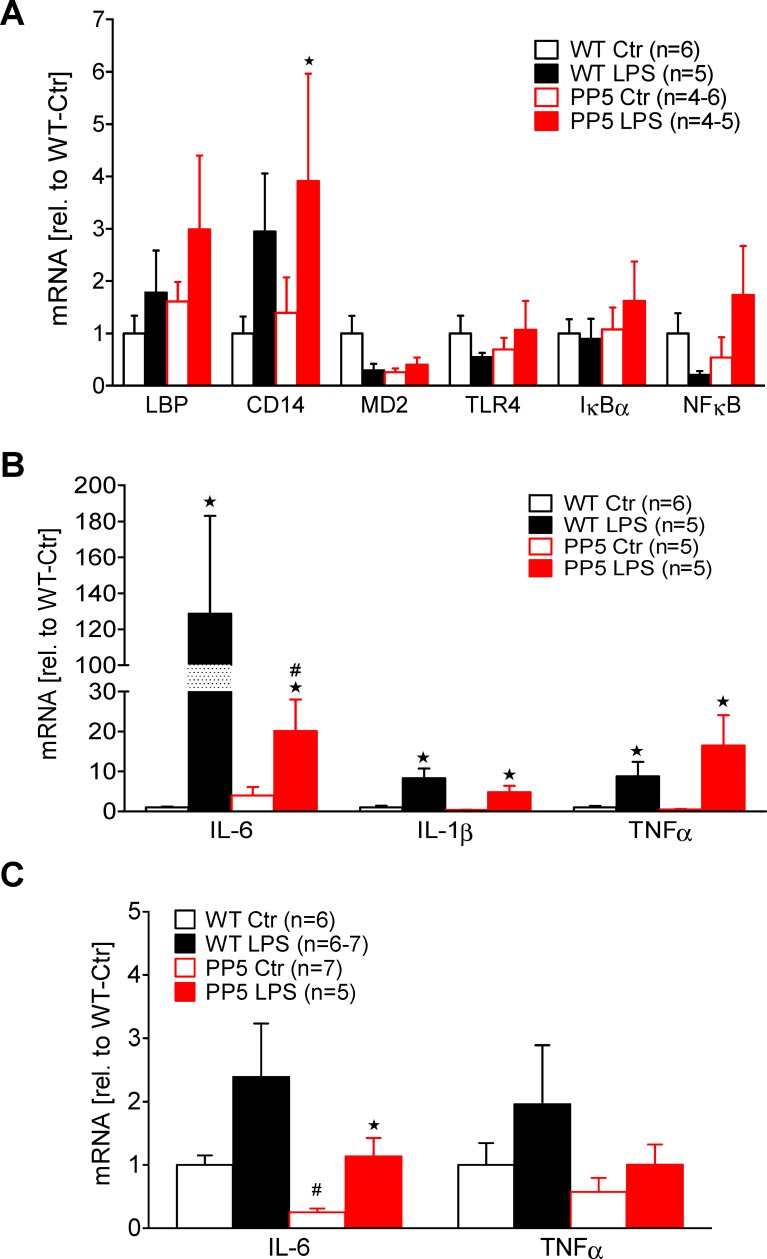
LPS-mediated gene expression. Cardiac gene expression analyzed by qPCR of wild type and PP5 overexpressing mouse hearts after intraperitoneal application of LPS (25 mg/kg) or NaCl solution as control (Ctr) after 7 h (**A, B**) and 3 d (**C**). (**A**) Expression of genes associated with the LPS signaling pathway and (**B, C**) expression of cytokines. Note that after three days, expression of cytokine mRNAs nearly reached basal values. Data were normalized to 18S RNA expression and WT by the 2^−ΔΔCT^ method. ★ p < 0.05 vs. Ctr, #p<0.05 vs. WT.

To get an idea on the mechanism how PP5 possibly can protect the heart, gene expression studies were performed. We analyzed the expression of selected genes of the inflammatory pathway from TLR4 to NF-κB by qPCR. Moreover, induction of cardiac cytokine expression was analyzed. The genes of the receptor complex, responsible for LPS detection, including TLR4, MD2, CD14, and LBP were not differently expressed in hearts of WT and PP5 mice under control conditions as well as after 7 h of LPS (Fig 3A). The expression of CD14 was increased by LPS in PP5 and tentatively in WT. Interestingly, MD2, a necessary part of the receptor complex, possibly was decreased in PP5 hearts and therefore may be responsible for the attenuated response to LPS. Because of the scattering these differences gained no significance. For IκB and NF-κB, important targets of the inflammatory signaling pathway (Fig 3A), the data are somewhat limited because of the scattering. Whereas IκB was unchanged, expression of NF-κB seems to be reduced in PP5 hearts under basal conditions reflecting a possible modulating influence of PP5 on this pathway. However, after LPS, NF-κB expression in PP5 hearts was highly variable between the animals, which make it hardly possible to evaluate any changes. The cardiac expression of the cytokines IL-1β, IL-6, and TNFα was clearly enhanced by LPS after 7 h in WT and in PP5 (Fig 3B). But in this first experimental group, the basal levels of cytokine mRNAs seem to be reduced in PP5 compared to WT even though without statistical significance (Fig 3B). Three days after LPS treatment (second experimental group), the mRNA expression of IL-6 was nearly normalized in WT but in PP5, although reaching WT levels, IL-6 was still enhanced compared to control whereas TNFα mRNA expression was normalized in both WT and PP5 (Fig 3C). It may be worthy of note that in PP5 hearts, basal cytokine mRNA levels (PP5-Ctr) were tentatively lower compared to WT-Ctr. This was the case for IL-1β and TNFα but not for IL-6 that was higher in group 1 (Fig 3B) but lower in group 2 (Fig 3C). Possibly, also the handling of the mice including control group (injections and anesthesia) may influence basal cytokine mRNA expression in any way, complicating the interpretation of basal cytokine expression.

**Fig 4:**
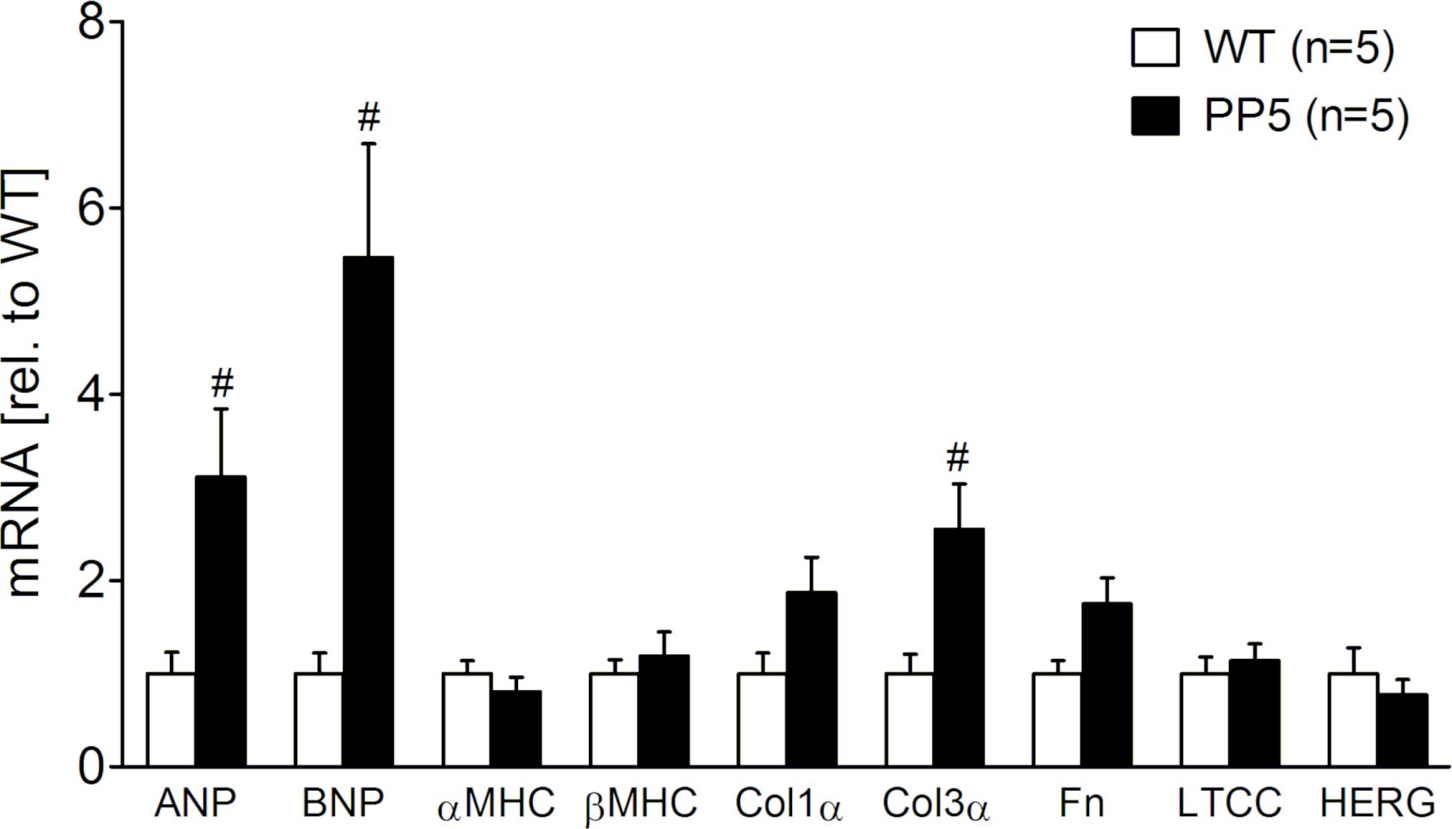
Basal mRNA expression. Basal cardiac gene expression of wild type and PP5 overexpressing mouse hearts analyzed by qPCR. Data were normalized to GAPDH expression and WT by the 2^−ΔΔCT^ method. #p < 0.05 vs. WT.

**Fig 5:**
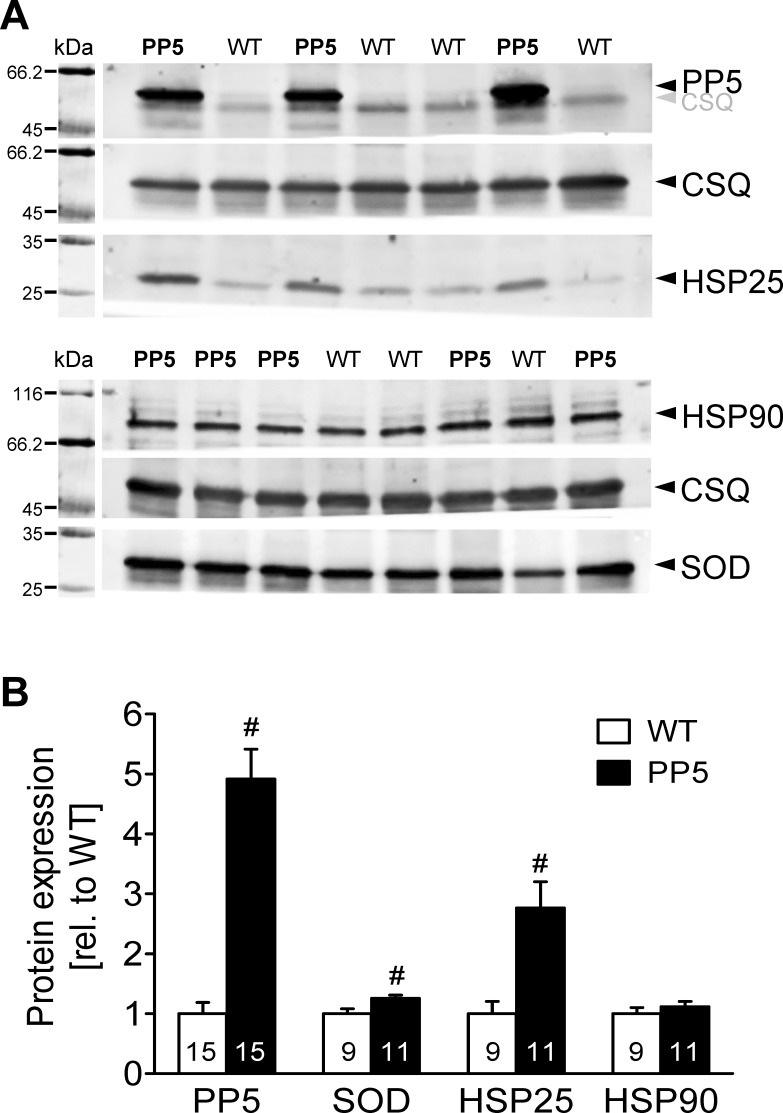
Basal cardiac protein expression. Basal cardiac expression of PP5, heat shock proteins 25 and 90 (HSP25, HSP90) and superoxide dismutase 2 (SOD) analyzed by Western blotting of wild type (WT) and PP5 overexpressing mouse hearts. (**A**) Representative original Western blots. Note that PP5 (~58 kDa) and CSQ (~55 kDa) were performed as double labeling experiment using two different wavelengths of the imager. But separation of signals was not complete; therefore, CSQ bands are also visible on the PP5 blot. The corresponding molecular weight markers are shown on the left side. (**B**) Quantification of Western blots normalized to cardiac calsequestrin (CSQ) as loading control and presented relative to WT expression. #p < 0.05 vs. WT.

Because neither the expression data in the acute phase after LPS treatment nor in the chronic phase gave a clear hint on the mechanism why PP5 hearts may be protected against septic cardiomyopathy, we looked at several other genes that can influence cardiac performance, independently of sepsis. On RNA level, we compared WT and PP5 hearts under basal conditions. We found an increased expression of ANP and BNP in PP5 (Fig 4). RNA expression of other genes, indicative for hypertrophy or fibrosis, like α and β-MHC, collagen 1 and 3, and fibronectin ranged from unchanged (α- and β-MHC) over tentatively increased (collagen 1 and fibronectin) to increased (collagen 3) in PP5 compared to WT (Fig 4). Conceivably, a PP5-dependent remodeling of the heart has been started also apparent as a reduced basal contractility, but RNA expression of two exemplary genes necessary for cardiac function, the calcium channel gene Cacna2 and the potassium channel gene Kcnh2, were unchanged (Fig 4). On protein level, by Western blotting, we detected a small but in our experimental group statistically significant increased expression of mitochondrial superoxide dismutase (SOD2) und HSP25 in PP5 hearts under basal conditions, indicative of an improved stress tolerance (Fig 5). Expression of another heat shock protein, namely HSP90 that is important for binding and targeting of PP5 was unchanged between WT and PP5 hearts (Fig 5). Other proteins involved in cardiac protein phosphorylation like CamKII, PP1 and PP2A including the catalytic subunit (PP2A-C), the regulatory A subunit (PP2A-A) and the regulatory G4-1 subunit, which is important as it recruits not only PP2A but also PP5 to the NF-κB pathway [5], were not altered (S1 Fig). Moreover, we also investigated expression of calcium regulatory proteins like calsequestrin (CSQ), SERCA, phospholamban (PLB), triadin, and junctin that were not altered (S1 Fig). Notable among them is that expression of CSQ was never different between WT and PP5 hearts when for example normalizing the antibody signals of the immunoblots to the color staining (Ponceau) of the blotting membrane. Therefore, we used CSQ expression as cardiac myocyte-specific housekeeping protein to normalize all protein expression data.

## Discussion

Sepsis is a life-threatening disease based on an inadequate immune response to a bacterial infection [30]. The pathophysiological mechanisms of sepsis-induced organ dysfunctions are multifactorial and largely unknown. Therefore, the only therapeutic options are a symptomatic treatment and an antibacterial therapy. The mortality of patients with a septic shock is in the range from 30% to 50% [31,32]. Furthermore, it is known that infectious-related cardiac dysfunctions can lead to heart failure. The so-called septic cardiomyopathy is diagnosed by reduced left ventricular contractility and reduced cardiac output together with a reduced vascular resistance [31,32].

Anti-inflammatory therapies have been rather unsuccessful for the treatment of sepsis. Therefore, explorations of the underlying mechanisms of septic disorders are currently in the focus for the development of new therapeutic strategies [33]. The contractility of the heart depends to a large extent on the phosphorylation state of regulatory proteins: for instance, very often an increased contractility is accompanied by increased phosphorylation of phospholamban. Moreover, gene expression is frequently regulated by phosphorylation. Much work has been done on studying protein kinases, less is known about their counterparts the protein phosphatases. Here, we focused on dephosphorylation as a regulatory mechanism in the heart, especially by PP5. Recently, we have demonstrated that PP5 is involved in the regulation of signaling and electromechanical coupling in the mouse heart [14]. We found basal protein expression of catalytic subunits of PP1 and PP2A unchanged in PP5 hearts compared to WT that implicates that basal change in cardiac contractility possibly was caused by enhanced PP5 activity. Nevertheless, there are still same gaps e. g. expressions of regulatory subunits of PP2A and/or PP1 or measurements of protein phosphatase activities especially after LPS are missing. In earlier works, others and we reported that human heart failure is accompanied by increased cardiac protein phosphatase activity [34,35]. Besides the known connection between phosphorylation of regulatory proteins and ischemic and idiopathic heart failure, a link was also found for sepsis. For instance, after LPS-application in wild type mice (CD1 background, like our mice), phosphorylation of the troponin inhibitor (TnI) was increased leading to a reduced Ca^2+^ sensitivity of the myofilaments which can in part explain a reduced ventricular performance by itself [36]. Similar effects were found in a rat model of sepsis [37]. In contrast, another work, using a rat model, demonstrated 4 h after LPS application a reduced PLB phosphorylation and an increased activity of serine/threonine phosphatases [38]. They found an increased activity of PP1 and PP2A. In hearts of patients, deceased due to sepsis, an increased mRNA expression of PP1 and PP2C was noted [39].

**Fig 6:**
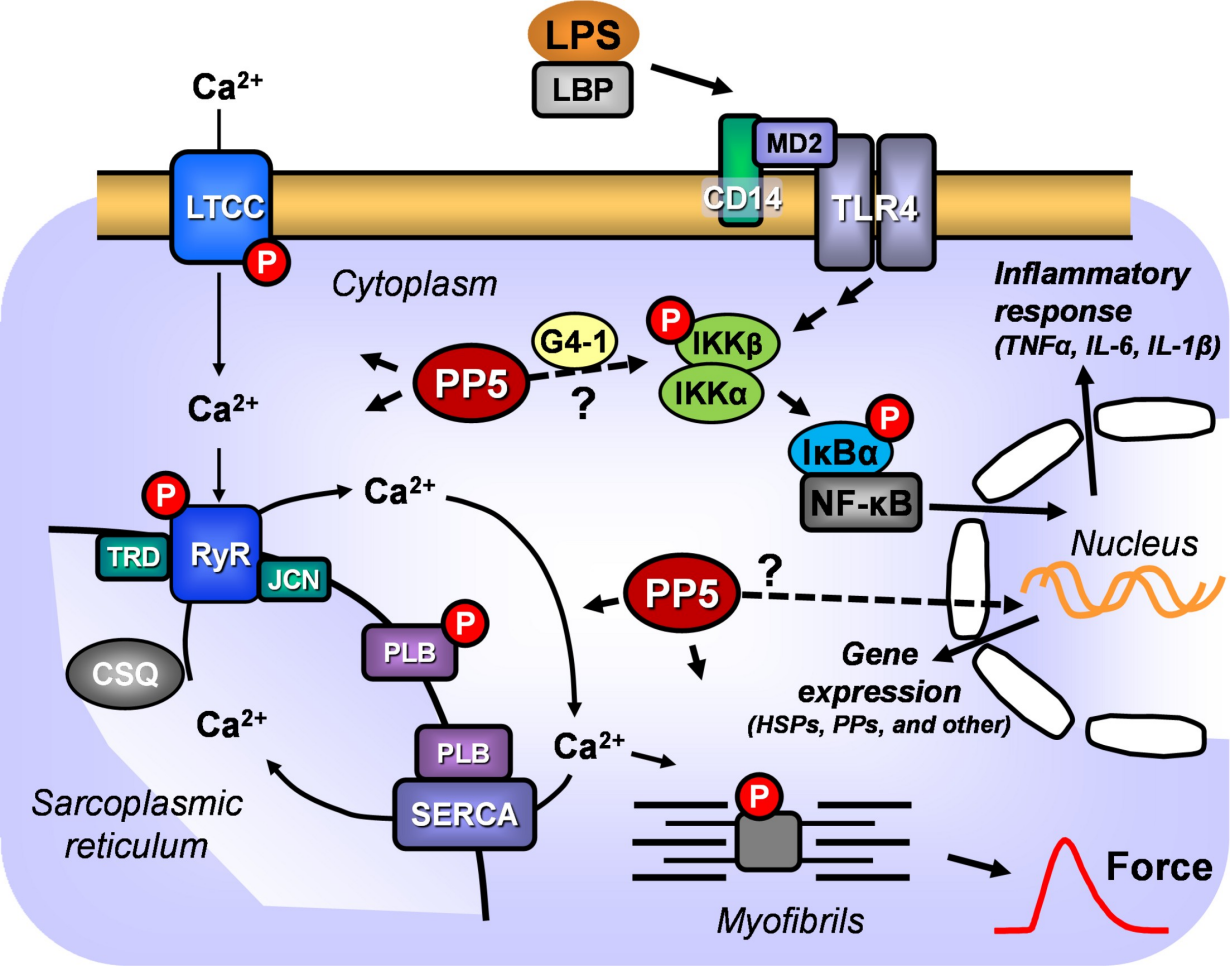
Possible role of PP5 in the cardiac myocyte. Schematic diagram illustrating possible roles of PP5 in the regulation of cardiac contractility and LPS signaling either by dephosphorylation of regulatory proteins or by influencing gene expression. PP5, protein phosphatase 5; LPS, lipopolysaccharide; LBP, LPS binding protein; MD2, myeloid differentiation factor 2; LTCC, L-type Ca^2+^ channel; JCN, junctin; G4-1, regulatory B” subunit of PP2A; IKK, inhibitor of kappa B kinase; IκB, inhibitor of kappa B; NF-κB, nuclear factor-kappaB; PLB, phospholamban; RyR, ryanodine receptor; SERCA, SR-Ca^2+^ ATPase; TRD, triadin; TLR4, toll-like receptor 4; TNFα, tumor necrosis factor α; IL, interleukin; P, phosphorylation; CSQ, calsequestrin; CD14, cluster of differentiation 14, that functions as an anchor protein.

A common inflammatory response, also found during sepsis, is the increase of cytokine expression like TNFα and others. In neonatal rat cardiac myocytes, TNFα was able to induce a reduction in TnI and PLB phosphorylation [40]. Moreover, there are data that PP2A activity can be increased by TNFα [41]. Using isolated cardiac myocytes from LPS-treated mice, a connection between PP2A activity and LPS-induced cardiac dysfunction could be demonstrated [42]. Following these data, an impact on several signaling pathways appears plausible. For one thing, regulatory proteins directly influencing cardiac contractility might be dephosphorylated, secondly, increased PP activity might change gene expression in the cardiac myocytes. The latter includes the activation of the transcription factor NF-κB that increases the expression of pro-inflammatory genes [43]. In this context, PP2A can act as negative or positive regulator of LPS-induced NF-κB activation dependent on the associated PP2A subunits [44]. Some regulatory PP2A subunits not only can recruit the catalytic PP2A subunit but also PP5. This includes the B” subunit G4-1/G5PR, which is responsible for negative regulation of NF-κB activation. The complex of G4-1 and PP5 interacts with and inactivates IKKβ leading to reduced phosphorylation of IκB and finally to reduced activity of NF-κB [5]. This mechanism may explain at least in part the weakened contractile responsiveness of PP5 transgenic hearts to LPS and the partially lowered cardiac cytokine expression in PP5 hearts, basal and after LPS. The scheme in Fig 6 illustrates how we imagine how PP5 possibly acts in the cardiac myocyte.

Another mechanism to protect the heart against LPS-induced damage might be an enhanced expression of protective proteins including chaperones like heat shock proteins (HSPs) or antioxidant enzymes like superoxide dismutases to reduce reactive oxygen species (ROS) that are produced in response to, for example, cytokines [45]. One important HSP that was found to reduce LPS-induced mortality in mice is HSP25 [46]. A common mechanism for upregulating HSP25 expression represents activation of the transcription factor heat shock factor-1 (HSF1) [47]. But HSF1 was found to be negatively regulated by PP5 [10]. Therefore, an alternative pathway for HSP25 upregulation should exist and was indeed described [46]. Application of riboflavin reduced cytokine expression likely through the HSP25 pathway but independently of HSF1 [46]. The exact pathway leading to increased HSP25 expression was not identified in this case but likewise, it may be the same that is involved in the HSP25 increase noted here, in PP5 overexpressing mice.

In a general context of ROS-dependent negative cellular effects, an influence of PP5 was described. For instance, in an Alzheimer disease model, a neuroprotective effect of PP5 against cell death induced by ROS generation was described in rat cortical neurons [48]. To best of our knowledge, a direct connection between SOD2 expression and PP5 has not been described so far.

In conclusion, we suggest that several signaling pathways are modulated by PP5 (Fig 6). First of all, signaling pathways leading to cardiac remodeling and reduced contractility, e. g. Ca^2+^ regulation, are modified. Increased expressions of natriuretic peptides and extracellular matrix proteins as well as reduced basal contractility are indicative for this assumption. These effects have been apparent by monitoring cardiac contractility *in vivo* (echocardiography). However, NF-κB signaling seems to be attenuated and moreover, together with an increased expression of HSP25 and SOD2, PP5 may act as positive regulator of stress tolerance in the heart.

### Limitations of the study

Nevertheless, our conclusions must be considered to be at least in part speculative. Some data are preliminary because, unfortunately, a direct interaction of PP5 with the described signaling pathways was so far impossible to determine. These are, for instance, the effect of PP5 on the activation of NF-κB and the pathway leading to an increased expression of HSP25 or SOD2 that are currently under investigation and will be the subject of further work. Moreover, several changes in protein and gene expression were not statistically significant complicating the interpretation of our results. The natural variability between experimental animals in general together with an unavoidable variability in handling them, possibly increases the risk of high scattering of experimental data. Especially RNA expression is known for a pronounced time-dependent fluctuation. In such case, high numbers of experiments could solve the problem, but this was beyond the scope of the study. A simple explanation would be that we just looked to the wrong genes. Again, this will be a subject of further work. One difficulty, in this context, is the absence of any cell membrane permeant specific and potent PP5 inhibitor or activator, as a pharmacological tool.

## Supporting information

S1 Table Primer sequences used for qPCR. (PDF)

S2 Table Primary antibodies used for Western blotting. (PDF)

S3 Table Organ weights of group 1 animals. (PDF)

S4 Table Organ weights of group 2 animals. (PDF)

S1 Fig Basal cardiac protein expression. (PDF)
