# Supplementary material for: Overexpression of protein phosphatase 5 in the mouse heart: Reduced contractility but increased stress tolerance – Two sides of the same coin?
Source: PLoS One. 2019 Aug 19;14(8):e0221289. doi: 10.1371/journal.pone.0221289 (PMC6699691; doi:10.1371/journal.pone.0221289)
Supplement: S1 Table — (PDF) [file pone.0221289.s001.pdf]

Table S1. Primer sequences used for qPCR.

Gene (accession number)	Forward primer	Reverse primer	Reference
18S RNA (NR_003278)	GTTGGTGGAGCGATTTGTCTGG	AGGGCAGGGACTTAATCAACGC	*
αMHC (NM_001164171)	GCTGACAGATCGGGAGAATCAG	CCCCTATGGCTGCAATGC	*
ANP (BC089615)	GTGCGGTGCCAACACAGAT	GCTTCCTCAGTCTGCTCACTCA	*
βMHC (NM_080728)	ACTCAAGCGGGAGAACAAGA	ACCTGGGACAACTCCAACTG	*
BNP (BC061165)	CCAGTCTCCAGAGCAATTCAA	AGCTGTCTCTGGGCCATTTC	*
Cacna1c (NM_009781)	TCACTGGCTCACAGAAGTGC	GATCTTCGTCTCCACCAGGA	*
CD14 (NM_009841)	GGCGCTCCGAGTTGTGACT	TACCTGCTTCAGCCCAGTGA	Bas et al., 2004
Col1α1 (NM_007742)	ACATGTTCAGCTTTGTGGACC	TAGGCCATTGTGTGTATGCAGC	*
Col3α1 (NM_009930)	TGGTAGAAAGGACACAGAGGC	TCCAACTTCACCCTTAGCACC	*
Fn1 (NM_010233)	TTAAGCTCACATGCCAGTGC	TCGTCATAGCACGTTGCTTC	*
GAPDH (GU214026)	CCAGCCTCGTCCCGTAGAC	ATGGCAACAATCTCCACTTTGC	Furlow et al. 2013
IκBα (U36277)	ATGAAGGACGAGGAGTACGAGCAA	TCTCTTCGTGGATGATTGCCAA	Wu et al., 2002
IL-1β (NM_008361)	TCGTGCTGTCGGACCCATAT	GTCGTTGCTTGGTTCTCCTTGT	Chen et al., 2006
IL-6 (NM_031168)	CCGGAGAGGAGACTTCACAG	TTCTGCAAGTGCATCATCGT	*
KCNH2 (NM_013569)	CGCAAGTTCATCATCATCGCTAA	AGGAAGCAGCTCCCATCTTT	*
LBP (NM_008489)	AGATCACACTACCGGACTTCAGCG	TTCCATTTGCCTCGGACACCGATG	*
MD2 (NM_016923)	GTCCGATGGTCTTCCTGGCGAGT	GCTTCTCAGATTCAGTCAATATGGG	Hajjar et al., 2012
NF-κB1 (M57999)	GAAATTCCTGATCCAGACAAAAAC	ATCACTTCAATGGCCTCTGTGTAG	Yamamoto et al., 2009
TLR4 (JX878359)	CTCTGCCTTCACTACAGAGAC	TGGATGATGTTGGCAGCAATG	Hajjar et al., 2012
TNFα (NM_013693)	CACACTCAGATCATCTTCTCAAAA	GTAGACAAGGTACAACCCATCG	*

*Primers were developed with the software Discovery Studio Gene v1.5 (Accelrys, Cambridge, UK)

References

Bas S, Gauthier BR, Spenato U, Stingelin S, Gabay C. CD14 is an acute-phase protein. J Immunol. 2004;172(7):4470-4479.

Chen YL, Huang YL, Lin NY, Chen HC, Chiu WC, Chang CJ. Differential regulation of ARE-mediated TNFalpha and IL-1beta mRNA stability by lipopolysaccharide in RAW264.7 cells. Biochem Biophys Res Commun. 2006;346(1):160-168.

Furlow JD, Watson ML, Waddell DS, Neff ES, Baehr LM, Ross AP, Bodine SC. Altered gene expression patterns in muscle ring finger 1 null mice during denervation- and dexamethasone-induced muscle atrophy. Physiol Genomics. 2013;45(23):1168-1185. doi: 10.1152/physiolgenomics.00022.2013

Hajjar AM, Ernst RK, Fortuno ES 3rd, Brasfield AS, Yam CS, Newlon LA, Kollmann TR, Miller SI, Wilson CB. Humanized TLR4/MD-2 mice reveal LPS recognition differentially impacts susceptibility to Yersinia pestis and Salmonella enterica. PLoS Pathog. 2012;8(10):e1002963. doi: 10.1371/journal.ppat.1002963

Wu T, Chen Y, Chiang SK, Tso MO. NF-kappaB activation in light-induced retinal degeneration in a mouse model. Invest Ophthalmol Vis Sci. 2002;43(9):2834-2840.

Yamamoto H, Omelchenko I, Shi X, Nuttall AL. The influence of NF-kappaB signal-transduction pathways on the murine inner ear by acoustic overstimulation. J Neurosci Res. 2009;87(8):1832-1840. doi: 10.1002/jnr.22018
