# Supplementary material for: Overexpression of protein phosphatase 5 in the mouse heart: Reduced contractility but increased stress tolerance – Two sides of the same coin?
Source: PLoS One. 2019 Aug 19;14(8):e0221289. doi: 10.1371/journal.pone.0221289 (PMC6699691; doi:10.1371/journal.pone.0221289)
Supplement: S2 Table — (PDF) [file pone.0221289.s002.pdf]

Table S2. Primary antibodies used for Western blotting.

Protein	Antibody (catalog number)	Vendor
PP5	Mouse monoclonal (611021)	BD Transduction Laboratories, Heidelberg, Germany
PP2A catalytic subunit	Rabbit monoclonal (ab32141)	abcam, Berlin, Germany
PP2A regulatory A subunit	Goat polyclonal (sc-6113)	Santa Cruz Biotechnology, Heidelberg, Germany
Calsequestrin	Rabbit polyclonal (SP5340P)	Acris Antibodies, Herford, Germany<br>(now available from abcam (ab3516))
CamKII	Rabbit monoclonal (2048-1)	Epitomics, Burlingame, CA, U.S.A.
SOD2	Rabbit polyclonal (SPC-118C/D)	StressMarq Biosciences, Victoria, Canada
HSP25	Rabbit polyclonal (ADI-SPA-801)	Enzo Life Science, Lörrach, Germany
HSP90	Rat monoclonal (ADI-SPA-845)	Enzo Life Science, Lörrach, Germany
G4-1 (PPP2R3C)	Rabbit polyclonal (abx014869)	Abbexa, Cambridge, UK
Phospholamban (PLB)	Mouse monoclonal (A010-14)	Badrilla, Leeds, UK
SERCA	Mouse monoclonal	Kindly provided by L.R. Jones, Indianapolis, IN, USA
Triadin	Rabbit polyclonal	
Junctin	Rabbit polyclonal	
