# Supplementary material for: Overexpression of protein phosphatase 5 in the mouse heart: Reduced contractility but increased stress tolerance – Two sides of the same coin?
Source: PLoS One. 2019 Aug 19;14(8):e0221289. doi: 10.1371/journal.pone.0221289 (PMC6699691; doi:10.1371/journal.pone.0221289)
Supplement: S3 Table — (PDF) [file pone.0221289.s003.pdf]

Table S3. Organ weights of group 1 animals. Body weight before (basal condition) and 7 h after LPS or NaCl (control) application, heart weight and relative heart weight (heart weight body weight ratio) of experimental animals (group 1).

	WT – NaCl (n = 6)	WT – LPS (n = 6)	PP5 – NaCl (n = 6)	PP5 – LPS (n = 6)
Basal body weight (g)	39.6 ± 0.9	37.2 ± 2.6	37.4 ± 1.7	39.4 ± 2.4
Body weight after LPS/NaCl (g)	39.1 ± 0.4	36.0 ± 2.5	37.2 ± 1.5	38.6 ± 2.4
Heart weight (mg)	203.0 ± 8.3	179.5 ± 18.7	177.8 ± 12.2	202.5 ± 1.9
Relative heart weight (mg/g)	5.2 ± 0.2	5.0 ± 0.3	4.8 ± 0.3	5.4 ± 0.3
