# Supplementary material for: Overexpression of protein phosphatase 5 in the mouse heart: Reduced contractility but increased stress tolerance – Two sides of the same coin?
Source: PLoS One. 2019 Aug 19;14(8):e0221289. doi: 10.1371/journal.pone.0221289 (PMC6699691; doi:10.1371/journal.pone.0221289)
Supplement: S4 Table — (PDF) [file pone.0221289.s004.pdf]

Table S4. Organ weights of group 2 animals. Body weight before (basal condition) and 3 days after LPS or NaCl (control) application, heart weight, relative heart weight (heart weight body weight ratio) and spleen weight of experimental animals (group 2).

	WT – NaCl	WT – LPS	PP5 – NaCl	PP5 – LPS
Basal body weight (g)	35.0 ± 1.9	33.6 ± 2.6	34.3 ± 0.9	34.9 ± 1.5
Body weight after LPS/NaCl (g)	35.8 ± 1.9	30.1 ± 2.6 *	33.7 ± 0.8	30.4 ± 1.4 *
Heart weight (mg)	189.9 ± 15.5	186.0 ± 18.8	188.9 ± 8.2	199.1 ± 15.4
Relative heart weight (mg/g)	5.3 ± 0.2	5.9 ± 0.2	5.7 ± 0.2	6.5 ± 0.2 #
Spleen weight (mg)	90.0 ± 6.1	160.3 ± 17.7 #	94.2 ± 6.9	177.8 ± 6.9 #

* $p < 0.05$ vs. basal conditions; # $p < 0.05$ vs. NaCl
