# Supplementary material for: Overexpression of protein phosphatase 5 in the mouse heart: Reduced contractility but increased stress tolerance – Two sides of the same coin?
Source: PLoS One. 2019 Aug 19;14(8):e0221289. doi: 10.1371/journal.pone.0221289 (PMC6699691; doi:10.1371/journal.pone.0221289)
Supplement: S1 Fig — (PDF) [file pone.0221289.s005.pdf]

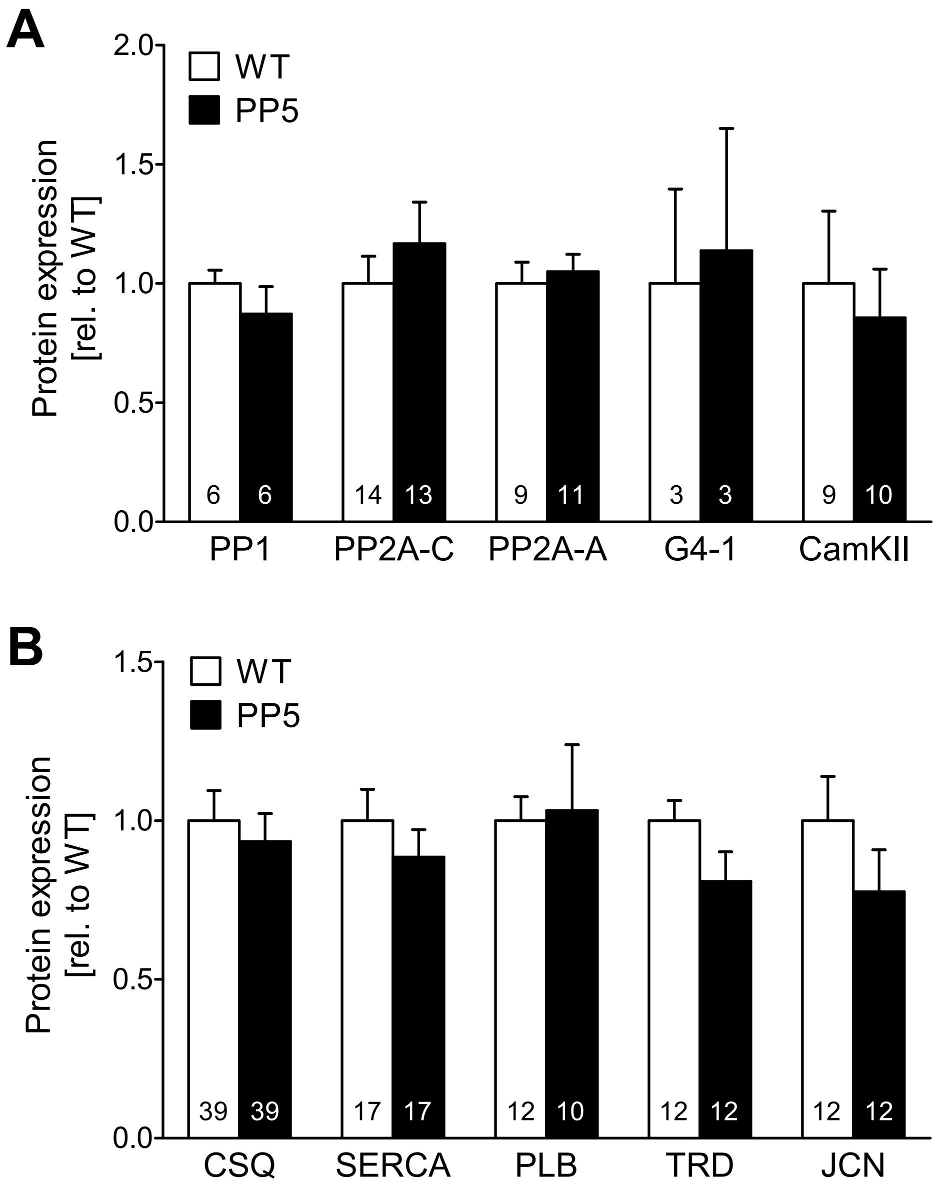

Fig S1. Cardiac protein expression. Basal expression of cardiac proteins of wild type (WT) and PP5 overexpressing mouse hearts analyzed by Western blotting. (**A**) Protein phosphatases and kinases, (**B**) Ca^{2+} regulatory proteins. Data were normalized to CSQ expression (of course with the exception of CSQ) and are presented relative to WT. Note that there was no difference in CSQ expression between WT and PP5 hearts. Numbers in columns are the numbers of analyzed hearts. G4-1, regulatory subunit targeting PP2A (and PP5) to the NF-κB pathway; CamKII, Ca^{2+} calmodulin kinase II; CSQ, calsequestrin; SERCA, sarcoplasmic reticulum Ca^{2+} ATPase; PLB, phospholamban; TRD, triadin; JCN, junctin.
